# Enlarged cross-sectional area in peripheral nerves in Swedish patients with hereditary V30M transthyretin amyloidosis

**DOI:** 10.1080/07853890.2023.2239269

**Published:** 2023-08-24

**Authors:** Sandra Arvidsson, Robert Eriksson, Intissar Anan, Victoria Heldestad

**Affiliations:** a Department of Surgical and Perioperative Sciences, Umeå University, Umea, Sweden; b Department of Clinical Microbiology, Umeå University, Umea, Sweden; c Clinical Neurophysiology, Umeå University Hospital, Umea, Sweden; d Department of Public Health and Clinical Medicine, Umeå University, Umea, Sweden; e Wallenberg Centre for Molecular Medicine, Umeå University, Umea, Sweden; f Department of Clinical Sciences, Umeå University, Umea, Sweden

**Keywords:** Hereditary transthyretin amyloidosis, nerve cross-sectional area, nerve entrapments, peripheral nerves, ultrasonography

## Abstract

**Introduction:**

In hereditary transthyretin amyloidosis (ATTRv), two different fibrillar forms causing the amyloid deposition, have been identified, displaying substantially cardiac or neuropathic symptoms. Neuropathic symptoms are more frequent in early-onset patients, whereas late-onset patients, besides cardiac symptoms, seem to develop carpal tunnel syndrome, more often. With ultrasonography (US) of peripheral nerves, it is possible to distinguish structural changes, and enlarged cross-sectional area (CSA). The main purpose of this study was, for the first time, to elucidate US of peripheral nerves in Swedish ATTRv patients at an early stage of the disease, and to evaluate possible early enlarged CSA.

**Material and methods:**

This prospective study included first visit data of 13 patients, aged 30–88 years, of which 11 with late-onset age. All had a positive V30M mutation. Eight men and six women (aged 28–74 years) served as controls.

**Results:**

Significantly enlarged CSA was seen in ATTRv patients for the tibial nerve at the ankle (*p =* .001), the sural nerve (*p <* .001), the peroneal nerve at the popliteal fossa (*p =* .003), and the ulnar nerve at the middle upper arm (*p =* .007).

**Conclusion:**

US of peripheral nerves could be a valuable tool in disease evaluation and could facilitate monitoring of disease progression.

## 1. Introduction

Hereditary transthyretin (TTR) amyloidosis (ATTRv), caused by a mutation in the TTR gene on chromosome 18, is a rare and fatal disease. Over 200-point mutations have been found worldwide [1], and in Swedish patients, the V30M mutation, is the most common one [2–3]. High prevalence is seen in both Sweden and Portugal, but differences in penetrance, age of onset and clinical manifestations of ATTRv are noted between endemic areas [4–6]. Unfolding of transthyretin (TTR) is required for fibril formation [7], which is manifested in several organs, such as the heart and peripheral nerves [8]. Two different fibrillar forms have been identified. Type A, with a mix of fragmented and full-length TTR, is most common in late-onset age patients and often manifest cardiac symptoms, whereas early onset patients seem to have type B (full-length TTR) to a greater extent, with primarily neuropathic symptoms [9]. Patients with late-onset age of the disease, also seem to develop carpal tunnel syndrome (CTS), a nerve entrapment of the median nerve at the wrist level, more often than early-onset patients [10]. However, CTS is not specific to the disease as CTS also is common for example in patients with diabetes, pregnant women, and workers in occupations with high physical exposure [11].

There is no curative treatment: until recently, liver transplantation has been the only option for slowing disease progression [12]. Today, there are new drugs available, for the treatment [13–17], which emphasize the importance of early diagnosis and early start of medication for optimal handling, and quality of life for each patient diagnosed with ATTRv. Since biopsy testing [18] is unsuitable for widespread routine screening, developing other clinical diagnostic procedures is of great value for these patients, especially in endemic areas.

Neurophysiological examination with combined nerve conduction studies (NCS) and needle electromyography (EMG) are both only used for the evaluation of larger myelinated fibers, and ATTRv patients with neuropathic symptoms is known to develop axonal loss of the peripheral nerves, which are initially seen as reduced/and later absence of amplitudes in sensory- and motor NCS [19]. Both NCS and EMG can be perceived as unpleasant for the patients, as weak electric current is given to the patients, and thin needles are inserted in muscles during the examination procedure. Patients with neuropathic symptoms initially often present impaired small-diameter nerve fiber function. It has been demonstrated that thermal quantitative sensory testing (QST) is a sensitive and useful method for the early detection of dysfunction in small-diameter nerve fibers [20], and a good complement contributing to an early diagnosis. Although thermal QST is dependent of patients’ awareness and participation, the method is reliable [21]. Still, new complementary clinical routine methods, are of significant importance, not only for these patients but also after the onset of unspecified neuropathic symptoms in endemic areas.

Ultrasonography (US) of peripheral nerves is not used to a large extent clinically for neurophysiological examinations today. Still, it should be used more since it is a non-invasive, and non-painful method. With the US it is possible to visualize structural changes of the peripheral nerves, detecting tumors or lipomas, and the method is a good complement during the evaluation of patients with suspected polyneuropathy (PNP) [22]. Studies have also shown that the US of peripheral nerves is clinically useful in entrapments, e.g. CTS [23,24], as this causes swelling and enlargement of the nerve proximal to the entrapment. In ATTR patients an enlarged cross-sectional area (CSA) might be caused by amyloid deposits in the endoneurium of peripheral nerves making the nerves more vulnerable to stretching around bone structures, i.e. medial epicondyle (ME) and at the caput fibulae at the knee, as well to compression in the carpal tunnel at the wrist [25]. In addition, an impaired nerve microcirculation could cause nerve damage and axonal loss in ATTRv [26]. There are previous US studies of peripheral nerves done in ATTRv patients with V30M, as well as other mutations [27–29], but none in Swedish patients.

The aim of this study was to evaluate the usefulness of US in peripheral nerves for the first time in Swedish ATTRv patients, and the purpose to measure the CSA at different distal and proximal nerve sites and assess possible early nerve entrapment.

## 2. Materials and methods

### 2.1. Subjects

This prospective pilot-study included first-time visit data from ATTRv patients, referred for a clinical routine specialist clinical neurophysiology evaluation at the Umeå University Hospital. The inclusion criteria for the patients were: verified ATTRv diagnosis, e.g. a verified positive transthyretin gene testing, as well as the finding of amyloid deposits in fat pad biopsy, or a positive DPD- (3,3-diphosphono-1,2-propanodicarboxylic acid) scintigraphy. Totally 13 patients, ten men and three women, aged 30–88 years, with a median onset age at 66 years were enrolled between November 2021 and April 2023. Eleven of the patients had TTR stabilizer treatment. All patients had a positive transthyretin gene mutation (V30M), see Table 1 for descriptive data as well as early clinical signs, and fibril types. The patients were also divided in relation to the age of onset of the disease; early onset (debut of clinical symptoms before 50 years of age) and late-onset (≥50 years). The ATTRv patients also underwent electrophysiological studies at the visit according to the local neurophysiological laboratory protocol to evaluate presumed PNP and/or CTS, including motor and sensory nerve conduction studies of the tibial, peroneal, sural, ulnar, and median nerves. Scoring for CTS and PNP were 0 = normal findings, 1 = mild, 2 = moderate, and 3 = severe.

**Table 1.** Descriptive data, and disease duration in 13 ATTRv patients (all with a V30M gene mutation, 11 with positive amyloid biopsy finding, two with positive ^a^DPD-scintigraphy).

Age	Gender	Duration (month)	Fibril type	Symptoms	Height (cm)	Weight (kg)
30	M	12	B	Numbness, tingling feet.	182	75.9
39	M	8	B	Numbness, tingling feet.	189	100
						
55	M	34	A	Numbness, tingling feet and hands.	178	111
56^a^	F	3	—	Tingling and numbness in feet	170	60.8
63	M	8	A	Numbness, tingling feet and hands.	182	70.4
64	F	2	B	Kidney failure.	165	68.5
66	F	21	B	Numbness, tingling feet. Alternating diarrhea/constipation.	169	78.5
68	M	2	A	Dyspnoea	183	108
70	M	84	B	Numbness and tingling feet and fingers. Kidney failure.	180	88
73	M	16	A	Numbness, tingling feet.	177	82
75^a^	M	13	—	Tingling feet, obstipation, dyspnea.	188	110
78	M	38	A	Tingling and numbness in feet and hands. Balance difficulties.	173	80
87	M	7	B	Balance difficulties.	180	80.6

^a^DPD (3,3-diphosphono-1,2-propanodicarboxylic acid).

A matched healthy control group was also included in the ultrasound study and consisted of 14 individuals, eight men and six women between 26 and 74 years of age with a median age of 55 years. They all were subjectively healthy (meaning, no for the individual, known neurological disease, CTS, or anything else that might affect peripheral nerves). Participation took place voluntarily and recruitment took place throughout students, medical staff or others. The controls were also divided in two age-groups (<50 and ≥50 years of age).

### 2.2. Ultrasonography of peripheral nerves

The US was performed by the same sonographer, and the same device was used throughout the study; a Canon Aplio A450 (© Canon Medical Systems Europe B.V., 2020), equipped with probe 18L7, model PLT-1204BT (© Canon Medical Systems Europe B.V., 2020). The frequency mode of the probe was set to 18 MHz.

**Figure 1.**
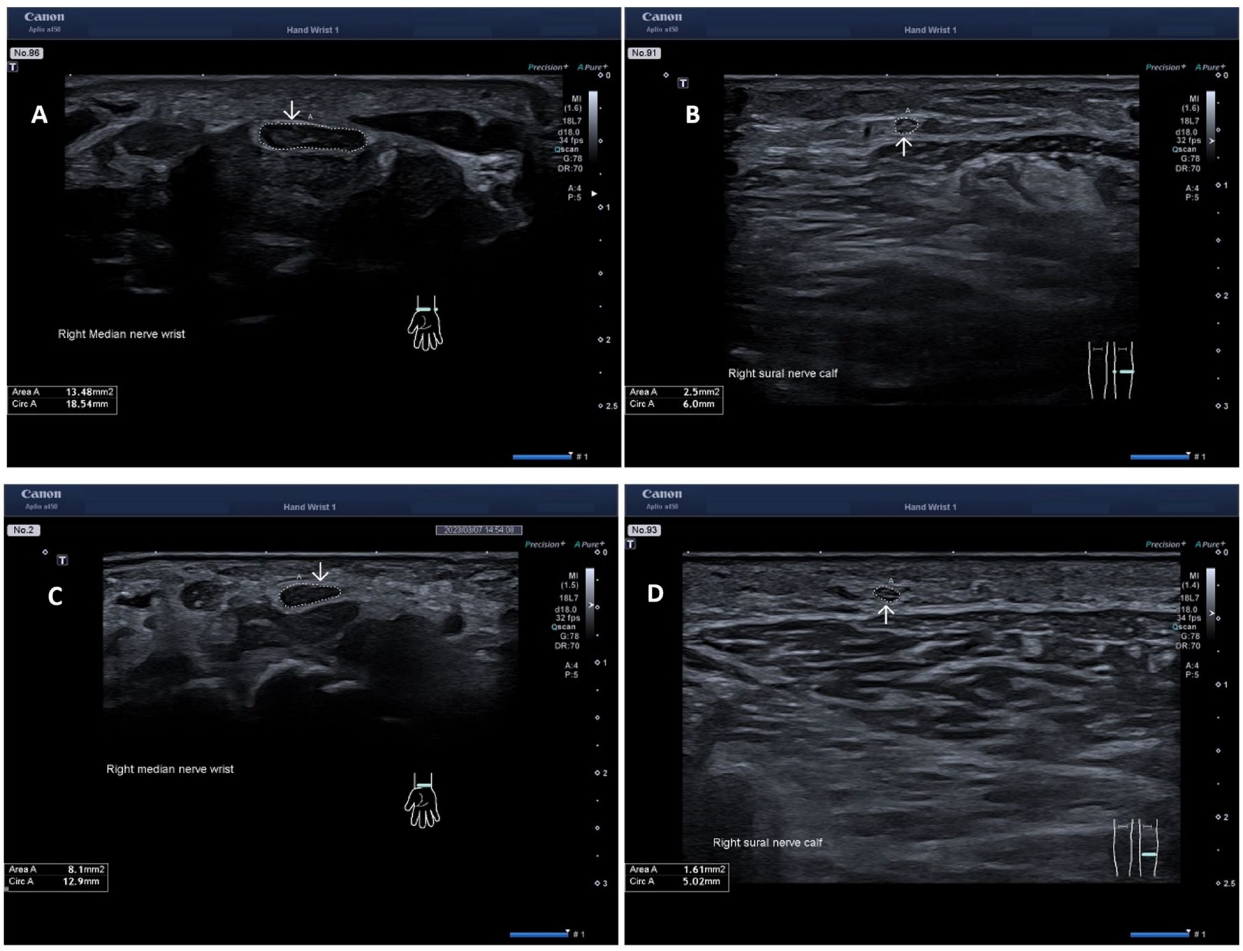
Ultrasound images in a patient with hereditary transthyretin amyloidosis (A) of the median nerve at the wrist, proximal to the carpal tunnel, (B) of the sural nerve, and in a healthy control (C) of the median nerve at the wrist, proximal to the carpal tunnel, (D) of the sural nerve. Arrows showing the cross-section area (CSA) mm^2^.

The following peripheral nerves were examined bilaterally: median, ulnar, peroneal, tibial, and sural. The nerves were examined in cross-section. The median nerve was examined at four sites; (1) at the wrist, proximal to the carpal tunnel, (MED_W_), (2) at one-third of the forearm (MED_F_), (3) at the elbow (MED_E_), and (4) at the middle of the upper arm (MED_U_). The ulnar nerve was also examined at four different sites: (1) at the wrist proximal to the pisiform bone (ULN_W_), (2) at the middle of the lower arm (ULN_F_), (3) at the median epicondyle (ULN_ME_), and (4) at the middle of the upper arm (ULN_U_). The peroneal nerve site was examined at the popliteal fossa (PER_PF_), 2–3 cm distal from the deviation from the sciatic nerve. The tibial nerve site at the ankle (TIB_A_) proximal to the medial malleolus, and the sural nerve site middle to the distal part of the calf. The eleven nerve sites were examined in cross-section with the probe 90° angle to the nerve. Depth and focus settings were adjusted for each nerve, depending on the current anatomical position. The most commonly used setting for depth was 2–3 cm with a focus exactly on the examined nerve/or just below the nerve to achieve the optimal image of the nerve’s epineurium. A minimum of two images were taken at each site for every nerve and the CSA was measured manually by following the epineurium of the nerve. An average area of the two technical best measurements were then calculated. The subjects were examined sitting upright in an adjustable chair while examining the median and the ulnar nerve. While examining the peroneal, tibial, and sural nerve the subjects lied in a prone position on their stomachs for easier access to the nerves. Figure 1, showing the CSA at the MED_W_ and the sural nerve in one patient and one healthy control.

### 2.3. Nerve conduction studies

Skin surface temperature was maintained >31 °C on the hand, and >30 °C on the foot, during all testing according to laboratory guidelines. Following parameters were measured to evaluate possible PNP and/or CTS; (1) sensory nerve amplitude potential (SNAP) of the median, ulnar and sural nerve, (2) the difference of distal sensory latency (DSL) between the median and ulnar nerve, (3) sensory conduction velocity (SCV) of the median (including fraction over the carpal segment), ulnar and sural nerve, (4) compound motor action potential (CMAP) of the median, ulnar, peroneal and tibial nerve, (5) distal motor latencies (DML) of the median, ulnar, peroneal and tibial nerve, (6) the difference of distal motor latency (DML) between the median and ulnar nerve, and (7) motor conduction velocity (MCV) of the median, ulnar, peroneal and tibial nerve. SNC was measured with antidromic technique, and ten averaged maximal nerve stimulations. Measurements from elicited waveforms were generally performed at the onset latency (except on the peak for sensory dig III over the carpal segment), and amplitude (baseline to peak). Supramaximal nerve stimulation recordings were done during MNC and measurements from elicited waveforms at the onset latency, and amplitude (baseline to peak).

Nerve conduction studies in the leg were always done bilaterally, and unilateral in the arm (dominant hand). If electrophysiological signs for CTS was found the contralateral arm was also conducted. All data was interpreted according to local laboratory reference values.

### 2.4. Statistical analysis

Statistical analyses were performed with commercially available spreadsheets and statistical programs (Microsoft Excel® and Jamovi^®^). Conventional descriptive, and non-parametric analyses were used. Shapiro-wilk was used to check for normality in controls. CSA (mm^2^) were described as mean, and range (minimal to maximum). Non-parametric analyses were used due to the low number of subjects. Mann–Whitney *U* test was used in testing differences between groups, and Kruskal–Wallis between testing > two groups. A *p-level* of *<*.05 was used to reject the null-hypothesis of no differences between samples.

### 2.5. Ethics

All participants received written information of the overall study design (including what happen to the participants clinical details and information that the study will be published. All participating provided both oral and written informed consent. The study followed the ethical guidelines of the Declaration of Helsinki. The study was approved by the Swedish Ethics Review Authority (2021/03826 and 2022/04551).

## 3. Results

### 3.1. Nerve conduction studies in ATTRv patients

All sensory and motor NCS were adjusted for the individuals age, gender, and length according to the local neurophysiological laboratory reference data. Two patients had early-onset of disease and eleven presented with late onset of symptoms. Both early-onset patients and three of the late-onset patients had normal sensory- and motor NCS bilaterally in the upper and lower extremities (e.g. no CTS or PNP). Two late-onset patients had normal sensory- and motor NCS bilaterally in the lower extremities (e.g. no PNP), but findings indicating mild CTS (bilaterally). One late-onset patient had a mild axonal, primary sensory PNP, and no CTS. Two late-onset patients had findings in the lower extremities indicating mild axonal sensory-motor PNP, and no CTS. Three late-onset patients had all moderate axonal sensory-motor PNP, along with one having mild CTS (right side), one moderate CTS (bilaterally), and one moderate CTS (left side), see Table 2.

**Table 2.** Age, gender, disease symptom duration, fibril type, and summary of nerve conduction findings in 13 patients with confirmed hereditary transthyretin amyloidosis.

Age	Gender	Duration (month)	Fibril type	PNP	CTS
30	M	12	B	0	0
39	M	8	B	0	0
					
55	M	34	A	0	0
56	F	3	—	0	0
63	M	8	A	0	1 (L + R)
64	F	2	B	0	0
66	F	21	B	1	0
68	M	2	A	0	1 (L + R)
70	M	84	B	1	0
73	M	16	A	1	0
75	M	13	—	2	1 (R)
78	M	38	A	2	2 (L)
87	M	7	B	2	2 (L + R)

CTS: carpal tunnel syndrome; PNP: polyneuropathy.

0 = normal findings, 1 = mild, 2 = moderate, 3 = severe, L: left side, R: right side.

### 3.2. Ultrasound

Ultrasound was performed, and the CSA was measured and evaluated bilaterally at all nerve sites in all controls. Due to limited examination time in three late-onset ATTRv patients, CSA was not measured and evaluated at all sites; in one patient (75 years of age), the CSA was not measured bilaterally at the TIB_A_, the PER_PF_ and the sural nerve, in one patient (63 years of age), bilaterally at the sural nerve and on the right sural nerve in one (88 years of age). The CSA in the ATTRv patients was measured bilaterally at all 11 sites (*n =* 22/22) at the MED_W,_ MED_F_, MED_E_, MED_F_, ULN_W_, ULN_F_, ULN_ME_, and ULN_U_. For the TIB_A_ and the PER_PF_ (*n =* 20/22), and for the sural nerve (*n =* 17/22).

#### 3.2.1. CSA in controls

No significant differences were found between sides at any of the tested nerve sites. Shapiro-wilk suggested an assumption of normality, except for the TIB_A_ (*p =* .016), the MED_E_ (*p =* .023), and the ULN_ME_ (*p =* .047). Between younger (< 50 years of age, *n =* 6) and older (≥ 50 years of age, *n =* 8) controls, no significant differences were found at any of the tested sites.

#### 3.2.2. CSA comparison between ATTRv patients and controls

**Figure 2.**
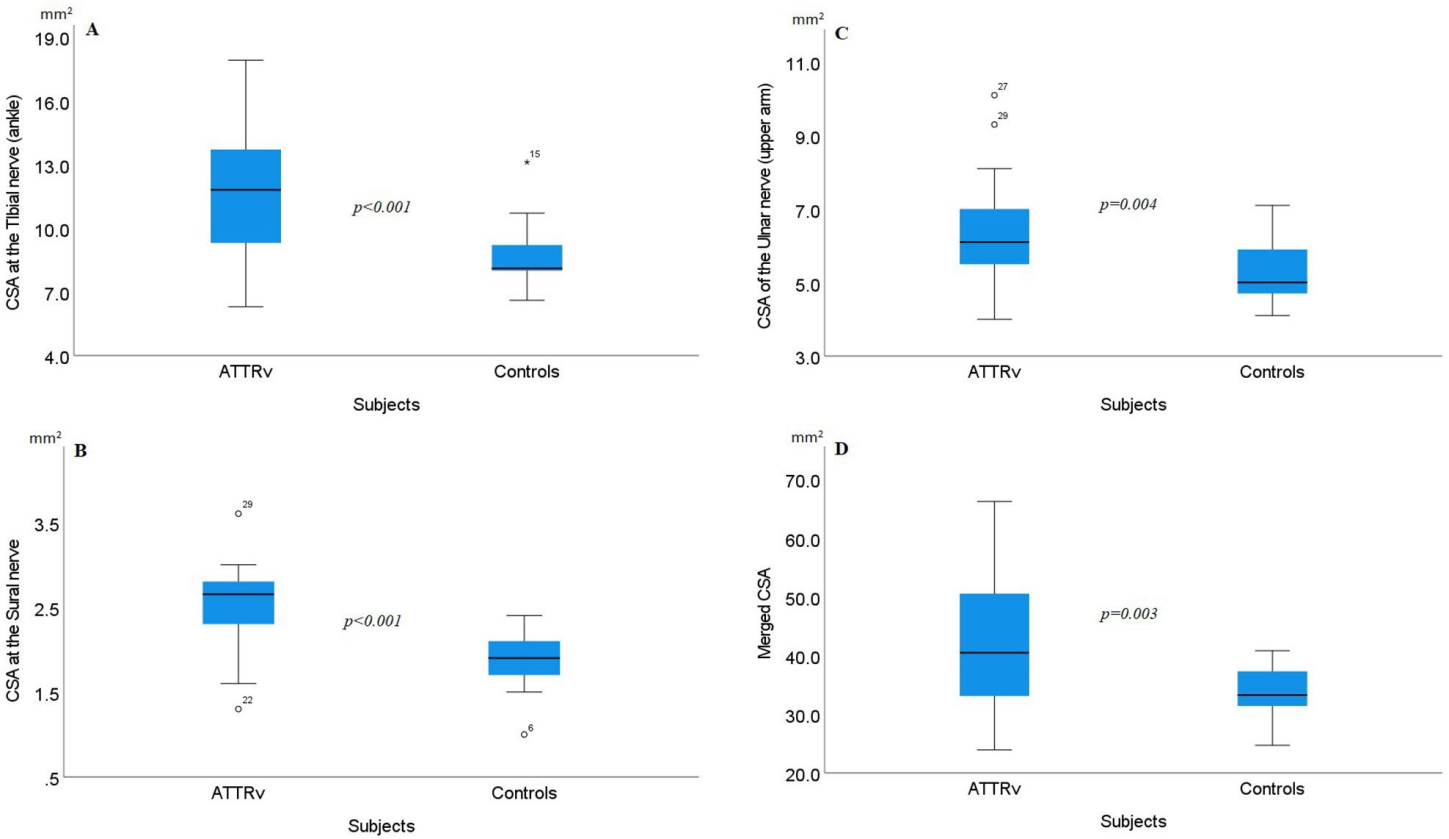
Boxplots showing cross-sectional areas (CSA) between 13 patients with hereditary transthyretin amyloidosis (ATTRv) and 14 healthy controls. The box shows median and percentage between 25 and 75. The whiskers showing the 95- percentage confidence interval (numbers indicating CSA data outside the 95% confidence interval). (A) CSA at the tibial nerve at the ankle, (B) CSA at the sural nerve, (C) CSA of the ulnar nerve (middle of the upper arm), and (D) Merged CSA = median CSA from the: tibial nerve at the ankle + sural nerve + peroneal nerve at popliteal fossa + median nerve at the wrist + ulnar nerve at the medial epicondyle.

In ATTRv patients, no significant differences were found between sides at any of the analyzed CSA nerve sites. There was no significant difference seen in weight (*p* = .124), height (*p =* .061) or BMI (*p =* .340) between patients and controls, but the control group was significantly younger (*p =* .048). Within the lower extremities a general significantly enlarged CSA was seen in patients at the sural nerve (*p <* .001), the TIB_A_ (*p* = .001) (Figure 2(A,B)) and the PER_PF_ (*p* = .003). Within the upper extremities, significant enlarged CSA was found at the ULN_U_ (*p* = .004), see Figure 2(C). A visual, but not significant larger CSA were noted at the MED_W_ (*p* = .275), the MED_F_ (*p* = .242), the MED_E_ (*p =* .063), the MED_U_ (*p* = .456), the ULN_F_ (*p =* .163), and the ULN_ME_ (*p =* .096). CSA at the ULN_W_ was almost equal between patient’s and controls (*p* = .959). At ULN_ME_, two presumed outliers were visually noted in controls, and if those were excluded also a significant enlarged CSA was seen in patients (*p =* .034). See Table 3 for CSA data (mean, range) in all subjects. Furthermore, a merged CSA with five different nerve sites (MED_W_ + ULN_ME_ + PER_PF_ + TIB_A_ + sural nerve) was evaluated, showing a significant enlarged CSA in patients (*p* = .003) (no presumed outliers excluded), see Figure 2(D).

**Table 3.** Ultrasound cross-sectional area data (mean, range) at 11 nerve sites, in 13 patients with hereditary transthyretin amyloidosis (ATTRv) and 14 controls.

Nerve sites	ATTRv mm^2^	*p-values*	Controls^a^ mm^2^
Median nerve at wrist	10.7 (5.5 − 20.5)^a^	.275	9.7 (6.1 − 15.7)
Median nerve at forearm	6.6 (3.4 − 11.5)^a^	.242	6.1 (4.3 − 9.7)
			
Median nerve at elbow	7.7 (4.4 − 15.2)^a^	.063	6.9 (4.4 − 10.8)
Median nerve at upper arm	8.9 (5.1 − 16.1)^a^	.456	8.0 (4.6 − 12.9)
Ulnar nerve at wrist	4.4 (2.3 − 7.0)^a^	.959	4.4 (2.8 − 6.0)
Ulnar nerve at forearm	5.6 (3.3 − 9.1)^a^	.163	5.0 (4.0 − 6.3)
Ulnar nerve at medial epicondyle	8.7 (4.2 − 22.4)^a^	.096	7.6 (4.8 − 13.8)
Ulnar nerve at upper arm	6.4 (3.71 − 10.3)^a^	.004	5.2 (3.5 − 7.5)
Peroneal nerve at popliteal fossa	6.7 (4.3 − 11.7)^b^	.003	5.3 (3.4 − 7.5)
Tibial nerve at the ankle	11.6 (6.2 − 18.0)^b^	.001	8.7 (6.1 − 13.9)
Sural nerve	2.5 (1.3 − 3.8)^c^	<.001	1.8 (0.8 − 2.6)

Total number of the 11 bilaterally measured nerve sites (left + right); number = ^a^22, ^b^20, ^c^17.

When comparing late-onset patients (≥ 50 years of onset age, *n* = 11) with controls older than 50 years of age (*n* = 8), also significantly enlarged CSA was found in patients at the sural nerve (*p <* .001), the TIB_A_ (*p =* .008) and the PER_PF_ (*p <* .001). Furthermore, within the upper extremities, significant enlarged CSA was found at the ULN_ME_ (*p =* .024) and the ULN_U_ (*p =* .027). No significant differences were noted at none at the other tested nerve sites.

#### 3.2.3. CSA and fibril types in ATTRv patients

The two patients with positive DPD scintigraphy were not fiber typed due to negative fat biopsy, and therefore excluded in the evaluation between fibril types.

Both patients with early onset age (*n =* 2) were men and had fibril type B. In patients with late-onset (*n =* 9), both fiber types were found. Five patients, all males, had fibril type A and four had fibril type B (two males, and two females). The CSA in patients with type A varied between: 2.2 − 3.8 mm^2^ (sural nerve), 9.7 − 15.4 mm^2^ (TIB_A_), 5.3 − 11.7 mm^2^ (PER_FP_), 9.5 − 20.5 mm^2^ (MED_W_)_,_ 4.7 − 11.5 mm^2^ (MED_F_), 6.5 − 15.2 mm^2^ (MED_E_), 6.3 − 16.1 mm^2^ (MED_U_), 3.0 − 6.2 mm^2^ (ULN_W_), 4.8 − 9.1 mm^2^ (ULN_F_), 7.4 − 22.4 mm^2^ (ULN_ME_), and 5.5 − 10.1 mm^2^ (ULN_U_). The CSA in patients with type B varied between: 1.3 − 3.2 mm^2^ (sural nerve), 7.3 − 18.0 mm^2^ (TIB_A_), 4.9 − 9.0 mm^2^ (PER_FP_), 6.8 − 12.4 mm^2^ (MED_W_)_,_ 4.5 − 9.6 mm^2^ (MED_F_), 4.4 − 10.1 mm^2^ (MED_E_), 6.2 − 13.5 mm^2^ (MED_U_), 2.9 − 6.0 mm^2^ (ULN_W_), 3.7 − 7.9 mm^2^ (ULN_F_), 4.2 − 13.4 mm^2^ (ULN_ME_), and 4.5 − 10.3 mm^2^ (ULN_U_).

**Figure 3.**
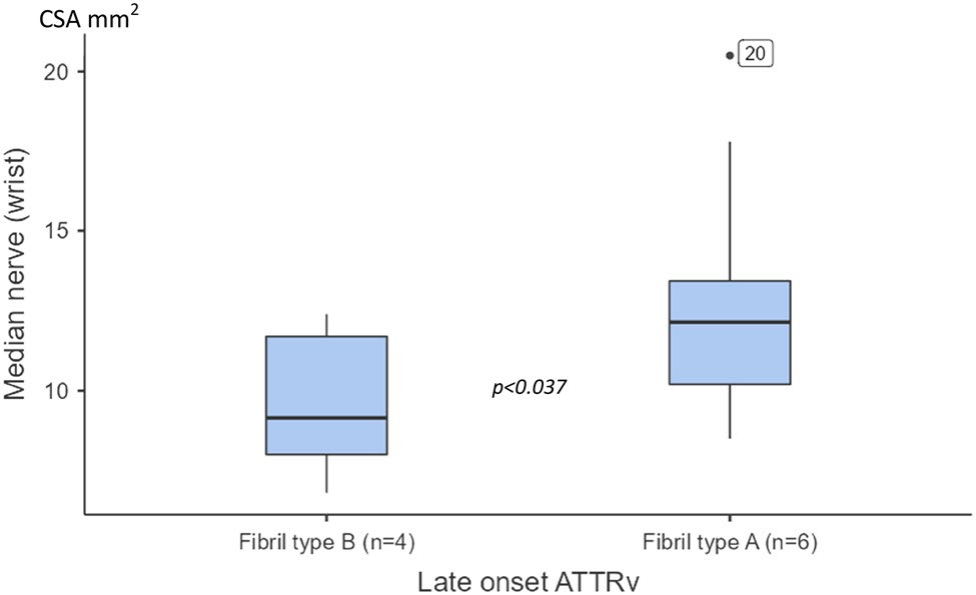
Boxplots showing cross-section areas (CSA) at the median nerve (wrist) between patients with late onset hereditary transthyretin amyloidosis (ATTRv), having fibril-type A and type B. The box shows median and percentage between 25 and 75. The whiskers showing the 95% confidence interval (numbers indicating CSA data outside the 95% confidence interval).

When including both early and late-onset fibril-typed ATTRv patients, those with fibril type A showed a significant larger CSA at the MED_W_ (*p =* .008) compared with those having fibril type B. No significant differences between fibril types were found for CSA at any of the other tested sites. When only late-onset patients were evaluated, also a significant enlargement was noted at the MED_W_ in patients with type A fibril composition (*p =* .031) (see Figure 3).

The most enlarged CSA at the MED_W_ was seen in the same patient, 17.8 mm^2^ on the left wrist and 20.5 mm^2^ on the right. Notable, the two oldest patients (one fibril type A and one type B) only had a CSA that varied between 6.8 and 10.5 mm^2^ at the MED_W_.

#### 3.2.4. Nerve conduction studies compared with CSA findings in ATTRv patients

**Figure 4.**
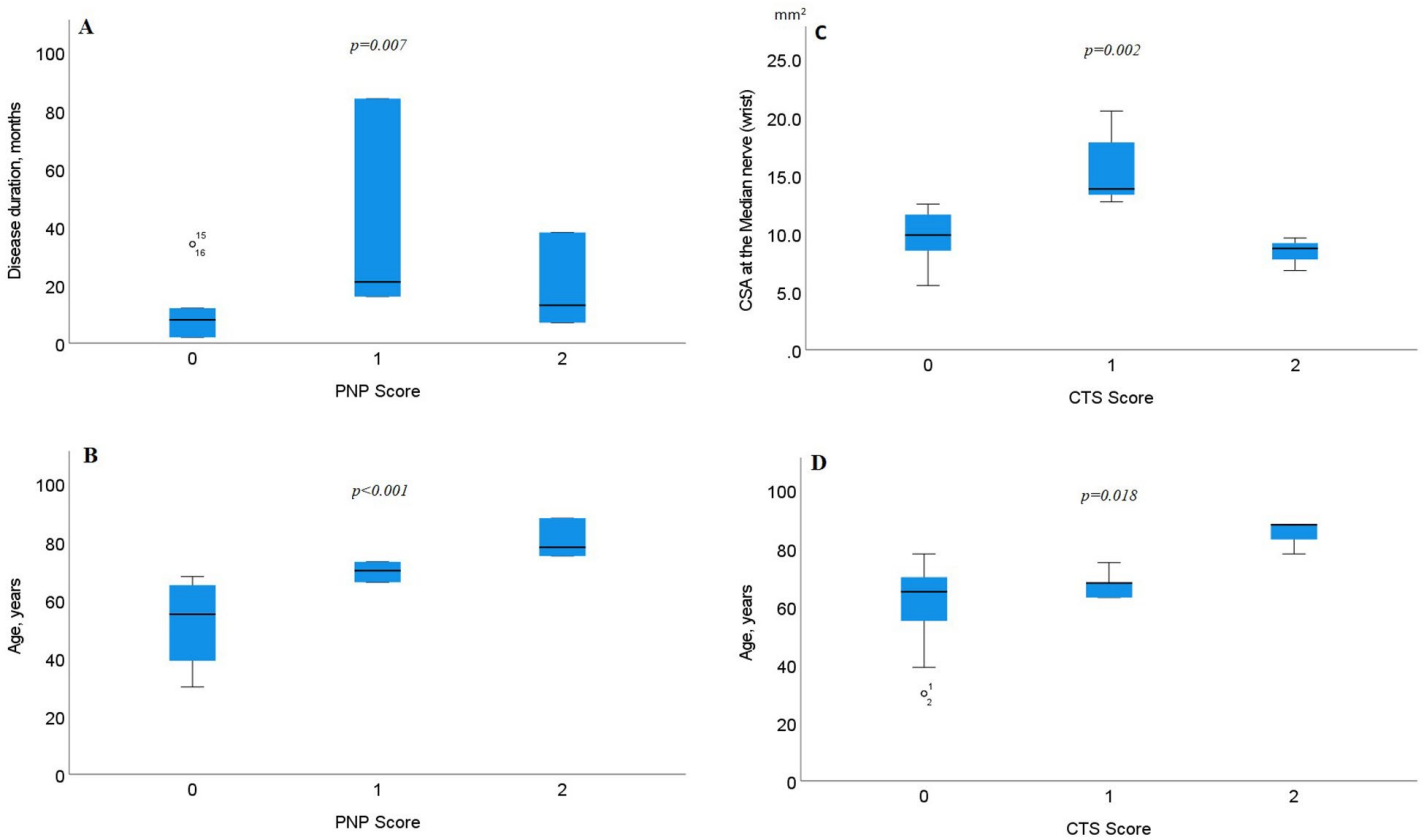
Boxplots showing differences with PNP scoring and (A) disease duration, (B) age of the patient, (C) CTS scoring and CSA at the median nerve at the wrist (MEDw), and (D) age of the patient. The box shows median and percentage between 25 and 75. The whiskers showing the 95% confidence interval (numbers indicating CSA data outside the 95% confidence interval).

There were no significant differences found between CSA in the sural (*p =* .868) or the tibial (*p =* .115) nerve compared with PNP scoring. However, significant differences with PNP scoring were found both with disease duration (*p =* .007) and the age of the patient (*p <* .001). Between CTS scoring and disease duration, no significant differences were found (*p* = .211). When comparing CTS scoring with the patients age, or the CSA at the MEDw, significant differences were found (*p* = .018 and *p =* .002, respectively), see Figure 4(A–D).

## 4. Discussion

This is the first study to evaluate the usefulness of ultrasonography of peripheral nerves in Swedish ATTRv patients and analyze findings according to fibril type. Our study also included patients with discrete symptoms, substantially tingling feet, and at a relatively early stage of the disease. Seven out of 13 patients did not show any electrophysical signs of PNP at all. We found significantly enlarged CSA in all three nerve sites within the lower extremities in ATTRv patients, distally, both at the sural and the tibial nerve at the ankle, as well as more proximal at the peroneal nerve. In the arm, we found significant differences at more proximal sites in the ulnar nerve, but not in the median nerve even though mean and maximal CSA was larger in patients. This study confirms previous findings, reporting thickening of peripheral nerves in ATTRv patients [27–29], but those studies differed in aspects that other mutation besides V30M were included. Our study is unique in one aspect since only those with a V30M mutation were randomly included.

Enlarged CSA in ATTR patients might be caused by amyloid fibrils deposits in the endoneurium making the nerves more vulnerable around possible entrapment sites. Moreover, an interfered microcirculation will lead to ischemic damage of the nerves. Amyloid deposits as well as impaired nerve microcirculation could be the cause an axonal loss in ATTRv [26], and prominent interstitial oedema and swelling of the nerves. In our study we focused on evaluating the CSA in five nerves, at three different sites in the lower extremities and eight different sites in the arms. Furthermore, we also focused on the wrist and elbow, which both are anatomical sites where nerves are vulnerable to damage [24]. Our initial idea was also to focus on caput fibulae at the knee during the US of the peroneal nerve, in order to examine three possible entrapment sites. However, it was technically difficult to acquire images with sufficient quality to evaluate, and therefore the popliteal fossa was evaluated throughout the study.

Both patients with early onset had fibril type B which is more common in patients with early onset age [9], along with more frequent autonomic neuropathic symptoms [30]. They both had discrete symptoms with numbness and tingling feet, but no electrophysiological signs of PNP or CTS. Since our study had a limited number of patients with early-onset disease, we could not draw any specific conclusions about the usefulness of US in peripheral nerves in younger V30M ATTRv patients. However, early onset age is less common in our endemic area [4,5]. Earlier studies have found that ATTRv patients with fibril type A often has late onset age [9] and that CTS are more common as well as cardiac involvement [10]. In our study, five late-onset patients had fibril type A, and four had type B, and we found that those with fibril type A had a significantly larger CSA at the MED_W_. Further, also a significantly enlarged CSA at the MED_W_ was seen within the 11 fibril-typed patients, regardless if onset age was evaluated. CTS is in the general population more common in women [11], and in our study all three women included were late-onset ATTRv patients, two with fibril type B and one in which fibril typing was not performed. With all this taken into account, our study support that enlarged CSA at the wrist, and that CTS in late-onset patients with fibril type A seems to be more frequent. Ehler suggests that a CSA at the wrist >10 mm^2^ would be critical [23]. With that in mind, 12 out of 22 late-onset wrists in our study should have had enlarged CSA, supporting a presumed CTS diagnose in eight out of 11 patients. Five patients showed electrophysiological signs of CTS (eight wrists).

Earlier studies have found enlarged CSA at multiple sites in ATTRv patients, but with difficulties drawing conclusions about these US findings, since mostly patients with different mutations than V30M were included [27–29]. Some reports no information at all about disease duration [27,29], whereas a disease duration between 1 and 18 years also has been reported [28]. Furthermore, there are some discrepancies between evaluated nerve sites [27–29]. Podnar et al. evaluated similar nerve sites, as those included in our study [28], however, only two of the patients out of 33 were V30M carriers. But studies have shown that disease severity and number of affected nerves seems to correlate [27,28]. Since enlarged brachial plexus has been found in symptomatic ATTRv patients with different genotypes [27,29], and disease progress evaluation [31], this nerve site would be interesting to evaluate in future studies in Swedish ATTRv patients. Another difference with our study and the previous once, is that in Sweden patients need to have abdominal fat amyloid deposits, or a positive DPD-scintigraphy besides being a gene carrier due to low disease penetrance. Previous reported studies [27–29,31], included carriers, with symptoms or electrophysiological findings of PNP as diagnose criteria which differs from Swedish patients diagnose criteria.

Further we wanted to evaluate another approach, to evaluate early clinical US findings in Swedish ATTRv patients. Our hypothesis was that a merged CSA at five evaluated sites could better visualize a general peripheral nerve involvement with larger CSA in ATTRv patients compared with healthy individuals. We found a significantly enlarged merged CSA in patients, which might support our hypothesis. However, more studies need to be performed in the future, both in healthy individuals to determine an optimal upper reference limit, and in early-onset patients. However, early onset is not frequent in our endemic area [4,5], which will make this very time-consuming to achieve.

Late-onset ATTR with a predominant neuropathic phenotype is occasionally mistaken for chronic inflammatory demyelinating polyneuropathy (CIDP), especially in non-endemic areas [32–34]. Findings of enlarged CSA by the US is not limited to ATTRv disease but also has been described as sensitive diagnostic finding in patients having CIDP [35]. Therefore, nerve US might not have the specificity to differentiate underlying causes to neuropathy. However, enlarged CSA, especially in endemic areas, should raise awareness for possible ATTRv disease and lead to further diagnostic tests. Extended studies are warranted to compare ATTRv with other axonal neuropathies at multiple sites, and elucidate the sensitivity of merged CSA for identification of ATTRv.

In our study, no significant difference in CSA was detected at the MED_W_ and MED_F_ between patients and controls. This contrasts with the findings of Salvalaggio et al., who found differences in multiple upper limb sites between ATTRv patients and asymptomatic gene carriers [29]. A possible explanation for this discrepancy is that our patient cohort was newly diagnosed and likely in an early stage of disease, and also with only included V30M patients, although our patients were randomly selected. Being that amyloid-related neuropathy initially affects lower extremities, larger CSA at the upper limb would likely be expected in patients with longer disease duration. This is also supported by our findings of significantly enlarged CSA in the lower extremities, at the sural nerve, the TIB_A_, and the PER_PF_. No significant differences between sides were found in CSA in our study within ATTRv patients. However, enlarged CSA was not found uniformly bilateral at all sites, especially at the arm, therefore we strongly recommend that the US should be performed on both sides. This is also supported by others [22,23]. But if the examination time is limited, we recommend the US at the lower extremities: the sural nerve, the TIB_A_ and the PER_PF_ as these three sites were the ones most significantly affected in our study.

Our study had some limitations with few controls and patients included, but our strength was that all our patients had a V30M mutation compared with others [28] with a similar number of patients but a variety of mutations included. Despite including few individuals, with mostly men included, we tried to gender, age, weight and length match the subjects. Despite that our controls were slightly significantly younger, we found significantly enlarged CSA both at all sites in the lower extremities, and at the ULN_ME_ and the ULN_U_ in the arms when we compared late onset ATTRv patients with controls older than 50 years of age. This confirms our findings of enlarged CSA in ATTRv patients. Further, all patients had a short disease duration, and the US was performed by the same sonographer.

Ultrasound of peripheral nerves has some limitations which must be considered. The peroneal nerve at the caput fibulae might get a pearly appearance when the nerve is going around the bone and deep into the tissue in the lower leg. Therefore, due to an elongated and unstructured appearance, it might be difficult to visualize and measure the epineurium. Also, as the nerve goes around the caput fibulae, one has to exactly follow the peroneal nerve anatomical structure in aspect to get an optimal 90-degree angle to receive the cross section of the nerve, and not a mixture between cross- and longitudinal section. Furthermore, sometimes the US image will form an eco-shadow precisely at the site where the peroneal nerve passes the caput fibulae. With these limitations in mind, the popliteal fossa was chosen instead of the caput fibula since that site was easier to examine. In future studies we are thinking of including both the PF and caput fibulae, and to look at the differences between those. Ultrasound of the ulnar nerve at the elbow is in general a very good method to use during neurophysiological examinations of the nerve since the structure of the nerve can easily be visualized, e.g. to see if the nerve is inflamed, if there is an entrapment, or if the nerve will move abnormally during dynamic testing of the elbow joint which is common problems [21]. However, the elbow is a difficult area to measure with the US and requires a lot of experience from the sonographer. The examiner needs to follow the nerves’ anatomical position around the ME with the probe to ensure that the probe always will be held at a 90° angle position towards the nerve to get a reliable cross-section measurement. Some individuals have a snapping ulnar nerve, meaning that during flexion of the elbow, the ulnar nerve will roll-over the ME from the medial side towards the lateral side and within most individuals the nerve will return to normal position during extension. Therefore, in cases with snapping nerve, the ulnar nerve will be measured during its normal position at the medial side of the ME at the elbow.

## 5. Conclusion

The present study emphasizes the importance of early introducing US of peripheral nerves in the clinical investigation of ATTRv patients as significantly increased CSA was found within patients, despite having short disease duration. This is of special interest in those with fibril type B, and younger patients with a V30M mutation, as they more often develop neuropathic [9] and autonomic [30] symptoms, as well as in late-onset patients as those with fibril type A seems to have more frequent enlarge CSA at the wrist [8,9]. Overall, an early diagnosis and early start of medication for the patients is important since the quality of life for each patient will improve [13–17], therefor additional tools for improving early diagnosis will benefit all patients with ATTRv. This is of importance since seven of our patients had no electrophysical findings of PNP, but we found significantly enlarged CSA in the lower extremities at all three nerve sites compared with controls. However, the US of peripheral nerves is of great value, not only in ATTRv patients, but in investigations of neuromuscular diseases, and nerve entrapment in general.

## Data Availability

Data available on request due to privacy/ethical restrictions.
